# Effect of Attachment and Child Health (ATTACH^TM^) Parenting Program on Parent-Infant Attachment, Parental Reflective Function, and Parental Depression

**DOI:** 10.3390/ijerph19148425

**Published:** 2022-07-10

**Authors:** Lubna Anis, Kharah Ross, Henry Ntanda, Martha Hart, Nicole Letourneau

**Affiliations:** 1 Owerko Centre for Children’s Neurodevelopment and Mental Health, Alberta Children’s Hospital Research Institute, Faculty of Nursing, University of Calgary, Calgary, AB T2N 1N4, Canada; lanis@ucalgary.ca; 2 Owerko Centre for Children’s Neurodevelopment and Mental Health, Alberta Children’s Hospital Research Institute, Department of Psychology, University of Calgary, Calgary, AB T2N 1N4, Canada; kharahr@athabascau.ca; 3 Faculty of Humanities and Social Sciences, Athabasca University, Athabasca, AB T9S 3A3, Canada; 4 Owerko Centre for Children’s Neurodevelopment and Mental Health, Alberta Children’s Hospital Research Institute, University of Calgary, Calgary, AB T2N 1N4, Canada; henry.ntanda@ucalgary.ca (H.N.); mhart@ucalgary.ca (M.H.); 5 Owerko Centre for Children’s Neurodevelopment and Mental Health, Alberta Children’s Hospital Research Institute, Departments of Pediatrics, Psychiatry, and Community Health Sciences, Cumming School of Medicine, University of Calgary, Calgary, AB T2N 1N4, Canada

**Keywords:** ATTACH^TM^, parenting intervention, parental reflective function, parent-child attachment, post-natal depression

## Abstract

High-risk families exposed to toxic stressors such as family violence, depression, addiction, and poverty, have shown greater difficulty in parenting young children. In this study, we examined the effectiveness of ATTACH^TM^, a 10–12 session manualized one-on-one parental Reflective Function (RF)-based parenting program designed for high-risk families. Outcomes of parent-child attachment and parental RF were assessed via the Strange Situation Procedure (SSP) and Reflective Function Scale (RFS), respectively. The protective role of ATTACH^TM^ on parental depression was also assessed. Data were available from caregivers and their children < 6 years of age who participated in five pilot randomized control trials (RCTs) and quasi-experimental studies (QES; *n* = 40). Compared with the control group, caregivers who received the ATTACH^TM^-program demonstrated a greater likelihood of secure attachment with their children (*p* = 0.004) and higher parental RF [self (*p* = 0.004), child (*p* = 0.001), overall (*p* = 0.002)] in RCTs. A significant improvement in parental RF (*p* = 0.000) was also observed in the QES within ATTACH^TM^ group analysis. As attachment security increased, receiving the ATTACH^TM^ program may be protective for depressed caregivers. Results demonstrated the promise of ATTACH^TM^ for high-risk parents and their young children.

## 1. Introduction

Parents who have histories of toxic stressors (e.g., family violence, depression, addiction) are at greater risk of being less sensitively responsive to their children and, in extreme cases, of losing custody of their children [1,2,3,4]. Toxic stressors also predict increased rates of developmental problems and physical health problems in children over the lifespan, and years of life lost to disability, resulting in health care system burden [5,6,7,8,9,10,11]. Moreover, parents with depression have shown not only lower sensitivity to their infants’ cues, but they may fluctuate between intrusive, punitive behaviors and passive withdrawal in interactions with their children [12,13]. These problematic or inept interactions increase the risk for insecure parent-child attachment [14,15], in which children cannot rely on their caregivers to provide a safe haven from distress and a secure base for exploration of the environment.

(Note: The term “parent” is referred to throughout this paper, this term reflects the fact that most literature on parenting and child development reviewed herein is on mothers, as parents. Thus, we use the term “parent” to be inclusive, and it refers to the primary caregivers who participated in the ATTACH^TM^ pilot studies). Parental Reflective function (RF), defined as a parent’s capacity to understand their own and their child’s thoughts, feelings, and mental states, likely underpins parents’ ability to be sensitive to their child’s needs and may buffer the negative effects of toxic stress on security of child attachment to their parent [15,16,17]. Thus, parental RF likely underlies sensitive responding by helping mothers mentally put themselves in the child’s place to imagine the child’s experience [16,17]. Additionally, parental RF may play a role in inhibiting problematic parenting interactions by helping parents become aware of their impacts on their child, thereby nurturing secure parent-child attachment [18]. In contrast, toxic stressors place parents at risk of negative and distorted representations of reality, frightening, or dissociated behaviors in interactions with children that impair parent-child attachment security [15]. Similarly, impairments in parental RF are associated with the development of insecure parent-child attachment and increased vulnerability for psychological disorders in childhood, adolescence, and adulthood [12,18,19,20]. It is therefore important to support parents to develop their RF in order to buffer the effects of toxic stress on their children [15,21].

### 1.1. Toxic Stress Undermines Attachment Security and Parental RF

Toxic stress affects children through multiple pathways, including biological embedding of adversities during sensitive developmental periods and cumulative social and economic damage over time [14,22,23,24]. While early adverse childhood experiences have the potential to influence health and development over the lifespan [22,25], impacts may be observed in early life with increased risk for insecure parent-child attachment [1,10,15]. Stressors are considered toxic to children’s development [8,9,10,26], as they function to reduce both parents’ sensitivity to their children’s needs and parents’ RF [15,27] and interfere with the formation of secure parent-child attachment necessary for healthy child development [1,28,29]. Consequently, families experiencing toxic stress are at increased risk of reduced RF and insecure attachment relationships [15].

### 1.2. Parental RF as a Buffer for Toxic Stress

Parental RF serves as a foundation for parent-child attachment [15,17,30]. It allows a parent to hold their child’s affect in mind, foresee their physical and emotional needs, adapt to their child’s needs and help their child to regulate themselves and thus create the context for secure attachment or conversely, insecure attachment [17]. A reflective parent has the capacity to make sense of their child’s behavior by considering their child’s thoughts, feelings, desires and intentions, for example, that the baby is crying because she is angry, or clings to the parent because she is scared [20]. Thus, secure parent–child attachment heavily depends on parental RF, necessary for a parent to appropriately perceive and accurately respond to their child’s cues that signal a need for soothing or exploration of the environment [17].

Of the toxic stressors that represent adverse childhood experiences, parental depression may be the most common [31]. Depression reduces parents’ capacity to be sensitively responsive to their children [32,33], with a negative impact on parent–child interactions and parental sensitivity linked to a host of health and developmental problems in children [34,35,36,37,38,39,40]. Taken together, these findings suggest the need for parenting interventions, specifically those focused on parental RF, that target the consequences of toxic stress on children [15,41,42,43,44,45,46,47,48,49,50,51,52,53].

### 1.3. Role of Parental RF in Parenting Interventions

Parenting interventions that focus on promoting parental RF have the greatest potential to improve the developmental outcomes of children exposed to toxic stress [15,41,42,43,44,45,46,47,48,49,50,51,52,53]. RF interventions were developed to address the challenges that attachment-based parenting interventions demonstrated in improving parenting sensitivity as RF was the missing component [54]. Randomized controlled trials showed that RF interventions effectively improved parenting sensitivity in the context of maltreatment [15,42,43,44,45,46,47,48,52,53]. To elaborate, extant parental RF interventions have predicted improvements in maternal–infant interaction, attachment security, and parental RF [15,42,43,45,51,52,53,55]. However, the limitations of these studies included small samples [15,52], longer length of the parenting programs [55], or homogenous samples e.g., drug addicts [42,46]. Moreover, to our knowledge, the effectiveness of a parental RF intervention on parental depression has not been explored to date.

### 1.4. Attachment and Child Health (ATTACH^TM^) Parenting Program

ATTACH^TM^ is a 10–12-week, manualized psychoeducational program with dyadic (caregiver, usually mother and child) and triadic (caregiver, usually mother, child, and co-parent) components, designed to promote RF in vulnerable families experiencing toxic stress [15,52,56,57]. The format consists of eight to ten face-to-face, one-on-one ATTACH™ program sessions with the parent and a RF facilitator and two to three face-to-face ATTACH™ program sessions with the parent, co-parent, and RF facilitator. One-on-one therapy consists of the facilitator focusing on building the therapeutic relationship and engaging in the RF approaches detailed below. Each one-on-one dyadic session unfolds in the same way, with the triadic sessions very similar in design; both providing maximum opportunities for the parent(s) to practice RF.

ATTACH^TM^ is designed to target parental RF to help the parents develop the capacity to think about mental states (thoughts, feelings, and intentions) and to consider how their own mental states might affect their children, and how their children’s mental states might have an impact on them. It aids the parents to better understand their children’s emotional needs, that may promote their children’s secure attachment to them [20]. To elaborate, the program helps the parents to identify and regulate their own emotional experiences that may help them regulate their behavior towards their children while keeping in mind their children’s mental states. The ability of being reflective in parenting is specifically relevant to mothers who are experiencing toxic stress since heightened distress (e.g., depression) and may lead to misattribution of mental states [20]. The short-term goals of ATTACH™ are to (a) engage caregivers in safe, supportive RF-focused sessions (b) engage caregivers in practicing RF (b) engage caregiver’s co-parenting support (e.g., child’s father, mother’s partner, mother’s friend, or family member) in supporting caregivers’ practice of RF. The long-term goals of ATTACH^TM^ are to (a) build the caregivers’ capacity for RF (b) support caregivers’ ability to be sensitive and responsive in interactions with child (and others) (c) support secure parent-child attachment, and (d) support healthy child development [57].

During the ATTACH^TM^ sessions, the facilitators work with the caregiver to bolster their RF skills by providing them an opportunity to practice their RF skills (please see [15,37,40,41] for more details). This is achieved by asking them to reflect on parenting moments, the way they perceive and reflect on their positive and negative experiences, so they become more aware of their own and child’s mental states to regulate their behavior [15,20]. If the child is not the focus, the facilitator brings the child into the caregiver’s mind. Stressful parenting moments, specifically those where the parent’s ability to reflect is compromised, are discussed in detail. By doing so, ATTACH^TM^ promotes the involvement of parents in a RF process by looking at different ways to perceive any stressful moment, rather than fixating on one particular way to view the content or situation [52,56,58]. These strategies also more likely help promote secure attachment [15]. The facilitator’s curious, inquisitive, non-judgmental behavior is considered crucial to the facilitator-client relationship, which in turn encourages the caregiver to remain actively engaged in a RF process [56].

### 1.5. Initial Three ATTACH^TM^ Pilot Studies

We previously reported findings from the first three ATTACH^TM^ pilot studies including two randomized control trials (RCTs) (*n* = 30) and one quasi-experimental (QES) (*n* = 10) with at-risk mothers caring for a child between birth and 3 years of age [15]. Attachment data were only collected at post-assessments in the first two pilots. We employed the IDEAS (Innovate, Develop, Evaluate, Adapt, Scale) method to guide the intervention testing for the first two pilots [15,52,57,59]. The IDEAS method allows for making adaptations to intervention design in the early stages on intervention testing [15,52,57,59]. Thus, the ATTACH^TM^ sessions were adapted from 12 to 10 based on the facilitators’ observations and participants’ feedback. ATTACH™ demonstrated positive impacts on maternal RF (d = 0.51–2.0) and maternal sensitivity (eta-squared = 0.30–0.47) in three small pilot studies (please see [15] for more details). Two trained ATTACH^TM^ facilitators (MH, LA) delivered the ATTACH^TM^ intervention.

### 1.6. Current Investigation

In this paper, we report findings from the five pilot studies including RCTs (pilot studies 3, 4 and 6) and QES (pilot studies 5 and 7) in an additional sample of 40 caregivers caring for a child < 6 years of age. The aims of these trials were to replicate and build upon the findings from the first three trials in a larger sample and specifically determine: (a) intervention impact on parent-child attachment, (b) intervention impact on parental RF, (c) if intervention-related change in parental RF predicted an improvement in parent-child attachment security, and (d) intervention impact on parental depression. We predicted that ATTACH^TM^ would improve parent-child attachment security, parents’ RF, and reduce parents’ depression. We also predicted that improvement in parental RF would associate with improved parent-child child attachment security.

## 2. Material and Methods

### 2.1. Overview

**Figure 1:**
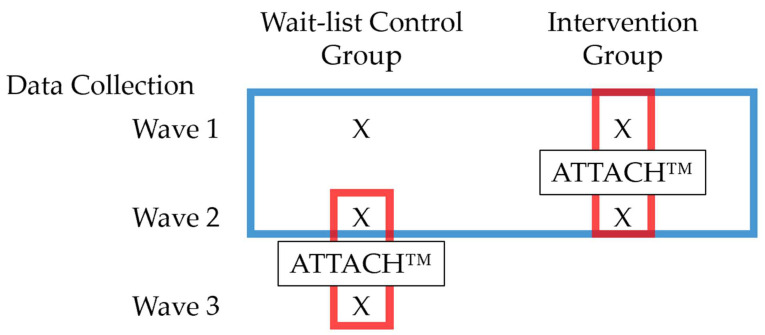
Schematic of data collection. Both intervention and wait-list control groups completed a baseline measurement (Wave 1). The intervention group completed the ATTACH™ Intervention between Wave 1 and Wave 2. Both the intervention and wait-list control groups completed an assessment after the intervention group completed the program (Wave 2). The wait-list control group then completed the ATTACH™ Intervention between Wave 2 and Wave 3. The wait-list control group completed another wave of assessment after the intervention was completed (Wave 3). The blue box indicates data points that were included in between-person analyses (intervention vs. control group, RCT). The red boxes indicate data points that were included in within-person analyses. (pre-to-post intervention change; QES).

The five pilots included RCTs (*n* = 40) and embedded QES (*n* = 16/40) and were conducted at three community agencies serving clients exposed to toxic stressors including poverty, family violence and depression. Two agencies were domestic violence shelters for women and children and the third agency addressed housing and education needs of low-income families. Caregivers [mothers (and grandparent)] and their children were enrolled in the study if they were able to read and write in English, had children < 6 years of age, and agreed to bring a co-parent. Co-parents (i.e., significant others of the parents’ choosing who were involved in parenting) were invited to participate in two sessions, and only data on relationship to the parent were collected from them. After completing baseline assessments, families were randomized to 10–12 sessions of manualized intervention. The QES were comprised of the control group derived from the pilot RCTs, after the RCTs were completed (please refer to Figure 1 for data collection waves). The study included a baseline assessment (Wave 1), a 10–12-week intervention, and a post-intervention assessment (Wave 2) for RCTs. Post-intervention assessments from RCTs were used as pre-assessments for QES (Wave 2). The wait-list control groups from the RCTs received the ATTACH^TM^ intervention later on and then completed the post-assessments (Wave 3). Trained facilitators delivered the intervention (MH, LA). To ensure intervention fidelity, facilitators complete a tool developed and refined during the first three RCTs (please see [57]). All of the procedures were approved by the Conjoint Health Research Ethics Board at the University of Calgary, Calgary, AB, Canada.

### 2.2. Sample, Recruitment and Randomization

Forty families were recruited at the participating inner-city agencies and a women shelter for victims of domestic violence by undertaking convenience sampling to recruit participants for ATTACH^TM^. The study participants were recruited through agency staff referrals, research assistant visits to group meetings, and flyers posted in the participating agencies. Interested parents were screened for eligibility by research assistants either in person or by telephone. Eligible parents met with a research team member to complete informed consent procedures. The compensation schedule for research participants was also explained. After the participants completed the baseline assessments, families were randomized to intervention (coded as 1) and control (coded as 0) groups by third party research staff based on a random assignment schedule created before recruitment [15,52,60,61]. The ATTACH^TM^ facilitators were not blinded to the group assignment; however, data coders were blind to group assignment.

#### 2.2.1. ATTACH^TM^ Pilot Study 3 (RCT)

We recruited 10 caregivers and children that were randomly assigned to intervention (*n* = 5) and control groups (*n* = 5) from one of the domestic violence shelters. Those caregivers were already partaking in another parenting program called Theraplay [62], that did not cover RF. Theraplay is designed to improve parent–child attachment and interaction quality, and children’s self-esteem by guiding the parent and child through playful games and developmentally appropriate activities [63].

#### 2.2.2. ATTACH^TM^ Pilot Study 4 (RCT)

A sample of 14 caregivers, each with 1 participating child less than 36 months of age, participated in the RCT at the inner-city agency serving low-income families. This sample was randomly assigned to an intervention (*n* = 7) and a control group (*n* = 7).

#### 2.2.3. ATTACH^TM^ Pilot Study 5 (QES)

The sample of pilot study 5 included the waitlist control group of ATTACH^TM^ pilot 4 (*n* = 7). All of the participants completed the ATTACH^TM^ program. Furthermore, all post-assessments from ATTACH^TM^ Pilot 4 were used as pre-assessments for ATTACH^TM^ Pilot 5.

#### 2.2.4. ATTACH^TM^ Pilot Study 6 (RCT)

A sample of 20 caregivers, each with 1 participating infant/young child less than six years of age, participated in the second RCT at a local shelter. This sample was randomly assigned to an intervention group (*n* = 10) and a control group (*n* = 10).

#### 2.2.5. ATTACH^TM^ Pilot Study 7 (QES)

The sample of pilot study 7 included the waitlist control group of ATTACH^TM^ pilot 6 (*n* = 10). All of the participants completed the ATTACH^TM^ program. Additionally, all post-assessments from ATTACH^TM^ Pilot 6 were used as pre-assessments for ATTACH^TM^ Pilot 7.

### 2.3. Participant Honoraria and Incentives

We used different strategies to enhance retention and minimize potential challenges to participation e.g., providing childcare with developmentally appropriate and friendly environment, participant honoraria, bus tickets, and gift baskets on completion of the program. Moreover, we organized the structure of financial compensation for the ATTACH^TM^ sessions in such a way that motivated them to complete the program. Payment for the sessions’ completion increased from $10–$20 per visit for the initial visits to $50–$55 per visit for the visits towards the end. We also provided developmentally appropriate toys to children to appreciate their participation in the study.

### 2.4. Demographic and Descriptive Measures

We collected data on caregivers’ and children’s demographic information and descriptive measure of toxic stress. We assessed social support with the Social Support Effectiveness Questionnaire (SSE-Q) that assesses women’s appraisal of partner social support in the three months prior to filling out the questionnaire [62]. The SSE-Q is a 25-item scale that asks respondents to provide a brief description of different types of partner social support (instrumental, informational, and emotional). Total scores range from 0 to 80, with higher scores indicating more effective support during the past 3-month period. The scale takes 5–10 min to administer and has an internal consistency of Cronbach’s alpha = 0.95 [62]. We examined adverse childhood experiences by employing Adverse Childhood Experiences (ACEs) Questionnaire, which is a 10-item tool that assesses adverse experiences before the age of 18 years [25]. ACEs exhibits excellent convergent validity and internal consistency (α = 0.88) [64], and higher scores indicate more exposure to ACEs.

### 2.5. Outcome Measures

#### 2.5.1. Parental RF

The Parent Development Interview (PDI) [65] is a 20-item semi-structured interview that takes approximately one hour to complete. The PDI is used to evaluate parents’ representations of their child and was digitally audio-recorded and transcribed for coding. Trained coders then employed the Reflective Function Scale (RFS) [2], that evaluates a person’s capacity to reflect on mental states in themselves and others in a communal context. The RFS scores PDIs on an 11-point scale that ranges from negative RF or anti-reflective (−1 = low RF, such as opaqueness of mental states) to full RF (9 = high RF such as awareness of mental states and ability to exhibit diverse view points) [65]. RFS scores are grouped in three subscales: Self score, Child score, and Overall score [65]. Scores of five or above, indicating higher parental RF, are typical in a normal population, while in a stressed or vulnerable population, scores of four or less, indicating lower parental RF, are typical [66]. MH coded the PDI interviews with the RFS, and to assess inter-rater reliability, coder agreement was assessed on 10% of PDI’s double coded with RFS by MH and the master RFS coder/trainer from The New School University, NYC, United States. They attained >80% for Overall scores, 86% for Self scores and 80% for Child scores. Furthermore, for our analysis, we calculated RFS change scores by calculating the difference of SSP Self, Child and Overall RFS scores between Wave 1 and Wave 2 for intervention and control group.

#### 2.5.2. Parent-Child Attachment

The Strange Situation Procedure (SSP; [1]) is the gold-standard assessment of attachment pattern in infants and young children. The SSP is an observational method that takes approximately 30 min to administer and three hours to code. In the SSP, a child and their parent enter an unfamiliar room and are introduced to a stranger prior to experiencing two separations and two reunions. During the procedure, children are exposed to mildly stressful events, specifically the entrance of an unfamiliar female adult (stranger) and two separations from their parents followed by reunions. The videorecorded SSP was coded with the ABCD [67,68] method for 9 to 20 month-olds and the MacArthur Preschool Assessment of Attachment (MAC) for children older than 20 months [69]. Past research has demonstrated that 8–10 week-long attachment-based intervention can alter attachment patterns in children [70].

Certified coders classified children into one of three organized attachment patterns [1] or a disorganized pattern [67,68]. Secure (Type B) children typically greet and seek contact with the caregiver upon reunion and return to exploration once settled. Avoidant (Type A) children are characterized by conspicuous avoidance of proximity to or interaction with the caregiver upon reunion. Resistant (Type C) children are perhaps most notable for their displays of ambivalence and anger with the caregiver in the reunion episodes. All child behavior within the procedure is noted, but the sequence of behavior during reunions is viewed as particularly informative [1]. Disorganized (Type D) infants, are characterized by disoriented behavior (e.g., wandering, confused expressions, freezing, undirected or contradictory movements when separated and reunited with the caregiver [69]. The SSP ABCD exhibits excellent internal consistency 0.78–0.88 [71], and interrater reliability from r = 0.53–0.98 [1,72,73].

For the SSP ABCD coding, MH was trained by Alan Sroufe’s Institute of Child Development at the University of Minnesota, and double-coded SSPs with Sroufe’s laboratory to attain 80% inter-rater reliability. For the SSP MAC coding, MH was trained by Bill Whalen of the University of Virginia, double coded SSPs with Whalen’s laboratory and attained 80% inter-rater reliability. MH also double coded SSPs one month apart and attained 100% intra-rater reliability.

An attachment improvement variable was calculated. For our analysis, dyads were coded as 1 if improved/remained secure or coded as 0 if insecure/not improved. For instance, the coders coded a child as improved or remained secured when a child was coded as A at baseline but coded as B after receiving the ATTACH^TM^ intervention (1). We did not have sufficient data to examine disorganized attachment pattern.

#### 2.5.3. Parental Depression

This was assessed by employing two different measures of depression. In pilots 3 and 4, the Edinburgh Postnatal Depression Scale (EPDS) [74] was administered. In pilots 5–7, the Centre for Epidemiologic Studies Depression Scale (CES-D) Questionnaire [75] was administered to assess depression as per the recommendations of the funding agency, the Harvard Frontiers of Innovation. The EPDS is a 10-item self-administered questionnaire measuring parental depression and involves a one week recall of symptoms. The EPDS correlates well with other measures of depression [12] and has excellent internal consistency, with Cronbach’s alpha = 0.87 [74]. Higher scores are often used to identify individuals who have more symptoms of depression [74]. The EPDS has a clinical cut-off value of 13, which is associated with clinical diagnosis of major depressive disorder [74]. The CES-D is a self-report tool consisting of 20 questions inquiring about individual’s level of psychological or mental distress [75]. Each item inquires about the frequency of symptoms of psychological or mental distress experienced within the past week. The range of scores is from 0 to 60, with higher scores indicating a higher level of psychological distress, with a clinical cut off of 16 or greater [75]. For analytical purposes, the scores attained from both measures of depression (EPDS and CES-D) were categorized into high and low depression scores, based on the clinical cut-offs. The scores were further categorized into remain depressed/improved e.g., if the caregivers’ depression scores did not improve post-intervention, they were classified as remain depressed whereas if their depression scores improved post-intervention, they were classified as improved.

### 2.6. Data Analysis

Demographic and descriptive characteristics of the sample are presented in Table 1. To test whether groups were similar with respect to baseline characteristics, independent *t*-tests were conducted (see Table 2).

Again, the pilot projects were a mix of RCTs and within-person designs (Figure 1), and different hypotheses were tested using data from different waves and/or treatment groups, depending on the nature of the question under investigation. First, to assess if the ATTACH^TM^ intervention improved parent-child attachment security relative to wait-list controls, between-group comparisons between intervention and control groups using Wave 1 and Wave 2 data were conducted (Figure 1). Attachment improvement from Wave 1 to Wave 2 was assessed using a binary logistic regression that included treatment group (intervention, wait-list control) as the predictor, controlling for baseline PDI-rated Overall RF scores (Wave 1). Baseline PDI-rated Overall RF was entered as a covariate because intervention and control groups differed with reflective function at baseline (Table 2). Significant odds ratios greater than 1.0 indicate that children in the intervention group demonstrated more improvements in attachment compared to wait-list controls.

Next to evaluate the effectiveness of ATTACH^TM^ on parental RF (Self, Child and Overall), between-group comparisons between intervention and wait-list control groups using Wave 1 and Wave 2 data were conducted using regression models that included treatment group (intervention, wait-list control) as the predictor, and controlling for baseline PDI-rated Overall RF scores (Wave 1). Separate regression models were conducted to evaluate improvements in PDI-rated Self, Child, and Overall RF scores. Significant odds ratios greater than 1.0 indicated improvements in RF scores (Self, Child and Overall) in the intervention group as compared to wait-list controls.

To examine if completing the ATTACH^TM^ intervention predicted improved PDI-rated RF score (Self, Child, Overall), within-person comparisons were conducted by using paired-samples *t*-test to see if PDI-rated RF post-intervention scores were significantly different from RF pre-intervention (*n* = 40).

To assess if the intervention-related change in RF (Self, Child, Overall) predicted improvement in parent-child attachment (within-person comparison; red boxes), Hayes conditional process modelling [76] was employed to test for mediation by using a logistic regression model (5000 bootstraps and 95% Confidence Interval) in SPSS version 24, controlling for baseline attachment and PDI-rated Overall RF scores (Wave 1) (*n* = 40); separate models for each PDI-rated RF (Self, Child, Overall) were employed.

To evaluate the effectiveness of ATTACH^TM^ on parental depression scores, cross-tabulations between remain depressed/improved depression scores were computed (*n* = 40).

## 3. Results

Table 1 represents the demographic and descriptive characteristics of the sample. At baseline, characteristics including demographics and descriptive characteristics including social support, ACEs, depression and the outcome of parent-child attachment were not significantly different between the two groups (see Table 2). However, the two groups were significantly different on PDI-rated RF Self score (*p* = 0.041) and PDI-rated RF Overall score (*p* = 0.040), favoring the intervention group.

### 3.1. Impacts on Parent-Child Attachment

Table 3 represents binary logistic regression results of between-groups comparisons to predict the effects of the ATTACH^TM^ intervention on improvement (or not) in parent-child attachment security (attachment improvement scores), after baseline PDI-rated Overall RF scores were controlled. A statistically significant difference between groups was observed for attachment improvement scores [*β* = 2.29, *p* = 0.004, 95% CI (0.02, 0.47)], favoring the intervention group (1 = Intervention group and 0 = Control group) and predicting that the intervention group is more likely to become securely attached.

### 3.2. Impacts on Parental RF and Depression

Table 4 presents multiple regression results of between-group comparisons to predict the effects of the ATTACH^TM^ intervention on PDI-rated RF change scores (Self, Child, Overall). A statistically significant difference between the groups was observed for PDI-rated RF Self scores [(*β* = 4.08, *p* = 0.004, 95% CI (0.40, 1.30)], PDI-rated RF Child Scores [(*β* = 3.82, *p* = 0.001, 95% CI (0.33, 1.32)], and PDI-rated RF Overall Scores [(*β* = 3.73, *p* = 0.002, 95% CI (0.33, 2.12)], favoring the intervention group. Table 5 presents the paired-sample *t*-tests comparing PDI-rated RF scores (Self, Child, and Overall), pre-intervention with post-intervention to assess within-group changes. There were significant differences in PDI-rated RF Self scores, *t*(40) = 4.43, *p* = 0.000), PDI-rated RF Child scores, *t*(40) = 5.97, *p* = 0.000), and PDI-rated RF Overall Scores, *t*(40) = 5.48, *p* = 0.000). Moreover, cross tabulations demonstrated a trend towards significance *X*^2^ (1, *n* = 40) = 3.48, *p* = 0.060) on parental depression scores as a result of intervention.

### 3.3. Intervention-Related Change in Parental RF and Parent-Child Attachment

Mediation analysis was employed to estimate the effect of group assignment (intervention = 1, control = 0) on attachment improvement scores (improved = 1, not improved = 0) mediated by PDI-rated RF Overall change, controlling for baseline attachment and PDI-rated RF Overall scores; Hayes conditional process modelling [76] was employed using a logistic regression model (5000 bootstraps and 95% Confidence Interval) in SPSS version 24. This modelling allowed conditional process analysis with a mediator i.e., the RF change score, while controlling for the PDI-rated RF Overall score at baseline and using attachment improvement scores as an outcome. Group assignment had a significant direct effect on attachment improvement scores [*β* = 2.88 (0.93), *p* = 0.002, 95% CI (1.05, 4.70)]. The indirect effect of group assignment to attachment improvement was non-significant [*β* = −0.09, 95% CI (−0.82, 0.69)]. Group assignment did not predict change in PDI-rated RF Self score [*β* = 0.42 (0.36), *p* = 0.235, 95% CI (−0.31, 1.15)]. Baseline attachment [*β* = −2.48 (1.39), *p* = 0.076, 95% CI (−5.22, 0.26)] and baseline PDI-rated RF Overall scores [*β* = −0.25 (0.56), *p* = 0.655, 95% CI (−1.36, 0.86)] were non-significant.

## 4. Discussion

In this paper, we reported results from the five pilot studies testing the efficacy of ATTACH^TM^, a 10–12 session manualized one-on-one parenting program focused on RF for high-risk families experiencing toxic stress. In both RCTs and QES, we found that ATTACH^TM^ significantly improved parent-child attachment security and parental RF in all areas including Self, Child, and Overall. To investigate if intervention-related change in parental RF predicted an improvement in parent-child attachment, we found that group assignment had a significant direct effect on attachment improvement scores, but did not predict a change in parental RF. A trend toward significant improvement in depressive symptoms was also observed for parents who received the ATTACH^TM^ program, suggesting possible protective effects. The implications of our results for clinical work and practice, parenting and attachment research, are discussed below.

### 4.1. Parent–Child Attachment

In our initial research, a trend toward a significant improvement in parent attachment security was observed (*n* = 30) [15]. More children (12%) were securely attached to their parents in the intervention group. However, significant differences were not noted in cross-tabulations. In our current investigation, ATTACH^TM^ intervention was associated with significant improvement in parent-child attachment security post-intervention with high-risk families. Others have also reported significant differences in parent-child attachment security in intervention versus control groups, with a 16% difference observed in the Minding the Baby trial [41,55,77]. Thus, our findings are supported by other research showing similar effects on parent-child attachment security. Moreover, it seems reasonable to expect that, as caregivers’ depression scores improve, this might have prevented the observed negative impacts of depression on attachment with their children [78].

### 4.2. Parent RF

Caregivers who took part in ATTACH^TM^ demonstrated a significantly higher capacity for RF after completion of ATTACH^TM^ sessions, indicating an enhanced ability to identify and understand of mental states in themselves and their children. The findings from this study are consistent with our previous research [15]. Overall, these findings demonstrate the promise and potential of relatively short-but-robust parental RF-based interventions for mitigating the effect of psychological challenges e.g., opaqueness of mental states [20,55,77] associated with less-than-optimal parenting. The results also highlight the importance of providing parental RF-based interventions to caregivers so that their mental health challenges such as depression, [79] can be targeted as they arise. Although our initial hypothesis that higher attachment security scores would predict higher parental RF scores was not supported by our results, these data may signal that, with a larger sample size, the results may be more pronounced.

### 4.3. Mental Health and Early Intervention Research

The comparison between intervention and control parents with respect to depression just missed statistical significance (*p* = 0.060). We observed the ceiling effects as the intervention group had lower scores on depression at baseline. This could have made a difference in depression scores harder to detect, as fewer of them were depressed at baseline. Although the ATTACH^TM^ intervention is not primarily designed to improve parental depression, the observed trends that were observed are encouraging and supported by existing research. Parental RF has been negatively affected by higher severity of depressive symptoms [79], meaning that high depression scores negatively impact the ability to mentalize [80] and a significant negative correlation was observed between low RF and parental sensitivity (*r*  =  −0.24, *p*  =  0.048) [79,81]. Both parental RF and depression may play related roles in undermining maternal sensitive responsiveness and may influence child attachment security [32,33,46,47].

Findings from current investigation and our past research [15] highlight the importance of interventions such as ATTACH^TM^ that target parental RF to enhance attachment security through psychoeducational education to promote healthy development in children from high-risk families [52,82,83]. As a quantifiable human psychological ability that is crucial for mutual interactions and functioning [84], RF’s relevance to attachment research, intervention and prevention will likely continue to grow [83]. Our findings along with the others’ [41,42,43,44,45,48,53,55,77,83,84] indicate that parental RF plays a central role in early years, as parents’ representations of their children can influence several domains of child development [15,56]. Nonetheless, the results from the study need to be verified with a larger sample size for the depression outcomes.

### 4.4. Strengths and Limitations

Our study has several strengths including a larger sample size as compared to our previous research and expert-level coding (MH) of the parent-child attachment and RF data. We were also able to employ state-of-the-science, age-appropriate measures of parent-child attachment (SSP MAC and ABCD coding) that enabled combination of children from 9 months to 60 months of age. Limitations include the necessity of combining measures of parental depression as a result of assessments via two different questionnaires that may have affected our ability to identify significance.

## 5. Conclusions

Toxic stress has a significant effect on parents in their ability to become sensitive and reflective in parenting, and for their children [14]. Intervention with high-risk families requires validated approaches that address the challenging health and development consequences of toxic stress. In our study, we pilot tested and validated on one such approach that is rooted in parental RF for improving parent-child attachment. In the next phase of our research program, staff members at partner agencies are being trained to deliver ATTACH^TM^ with fidelity, to test whether ATTACH^TM^’s efficacy sustains when it is delivered by staff members in a community setting.

## Figures and Tables

**Table 1:** Sample descriptive (*n* = 40).

Sample Descriptive RCTs (*n* = 40)		
Variables	Mean (SD)	Frequency (Percentage)
Age, Caregiver [years]	31.24 (6.06)	
Age, Child [Months]	31.08 (14.48)	
**Caregiver’s Ethnicity**		
Caucasian		18 (45.0%)
South Asian		2 (5%)
West Asian (Iranian, Afghan)		3 (7.5%)
Aboriginals/Natives		9 (22.5%)
African		6 (15%)
Latin American		1 (2.5%)
Chinese		1 (2.5%)
**Highest Level of Education**		
Less than High School		14 (35.0%)
High school		11 (27.5%)
Post-secondary education		15 (37.5%)
**Caregivers Marital Status**		
Married/Common in Law/Engaged		4 (10.0%)
Single/Separated/Divorced/Widowed		36 (90.0%)
**Caregiver’s ACEs for RCTs**		
Low < 3		19 (47.5%)
High < 4		21 (52.5%)
**Sample Descriptive QES (*n* = 15)**		
Variables	Mean (SD)	Frequency (Percentage)
Age, Caregiver [years]	30.41 (13.32)	
Age, Child [Months]	32.00 (6.25)	
**Caregiver’s Ethnicity**		
Caucasian		6 (40.00%)
Non-Caucasian		9 (60.00%)
**Highest Level of Education**		
Less than High School		6 (40.00%)
High school		5 (33.3%)
Post-secondary education		4 (26.7%)
**Caregivers Marital Status**		
Married/ Common in Law/Engaged		1 (6.7%)
Single/Separated/Divorced/Widowed		14 (93.3%)
**Caregiver’s ACEs for QES**		
Low < 3		8 (53.3%)
High < 4		7 (46.7%)

**Table 2:** Group Comparisons at Baseline (*n* = 40).

	Intervention Group (Mean, SD)	Control Group (Mean, SD)	*p*-Value
Age, Caregiver [years]	30.47 (5.94)	32.00 (6.25)	0.470
Age, Child [Months]	31.76 (15.94)	30.41 (13.32)	0.790
**Caregiver’s Ethnicity**	**Frequency (Percentage)**	**Frequency (Percentage)**	
Caucasian	9 (42.85%)	9 (47.36%)	
Non-Caucasian	12 (57.14%)	10 (52.63%)	0.781
**Highest Level of Education**			
Less than High School	5 (23.80%)	9 (47.36%)	
High school	6 (28.57%)	5 (26.31%)	
Post-secondary education	10 (47.61%)	5 (26.31%)	0.101
**Caregivers Marital Status**			
Married/Common in Law/Engaged	3 (14.28%)	1 (5.26%)	
Single/Separated/Divorced/Widowed	18 (85.71%)	18 (94.73%)	0.355
**Caregiver’s ACEs**	4.80 (3.08%)	3.74 (3.07%)	0.288
**Caregiver’s Social Support Scores**	45.45 (11.02%)	43.84 (12.36%)	0.670
**Caregiver’s Depression**			
>clinical cut-offs	17 (80.95%)	15 (78.94%)	
<clinical cut-offs	4 (19.04%)	4 (21.05%)	0.947
**Attachment Scores**			
Secure	3 (14.28%)	2 (10.52%)	
Insecure	18 (85.71%)	17 (89.47%)	0.561
PDI-rated RF Caregiver Scores	2.71 (0.87%)	2.10 (0.95%)	**0.041**
PDI-rated RF Child Scores	2.42 (0.87%)	1.94 (0.99%)	0.112
PDI-rated RF Overall Scores	2.57 (0.75%)	2.02 (0.86%)	**0.040**
Group Comparisons Post-Intervention (*n* = 40)			
**Caregiver’s Depression**			
>clinical cut-offs	10/20 (50%)	15/19 (78.94%)	0.060
<clinical cut-offs	10/20 (50%)	4/19 (21.05%)	
**Attachment Improvement Scores**			
Secure	16/21 (76.19%)	5/19 (26.31%)	**0.004**
Insecure	5/21 (23.80%)	14/19 (73.68%)	
PDI-rated RF Self Score	3.80 (1.14)	2.39 (1.27)	**0.001**
PDI-rated RF Child Score	3.57 (0.99)	2.05 (1.14)	**0.000**
PDI-rated RF Overall Score	3.65 (1.01)	2.23 (1.15)	**0.000**

Bold: Statistically significant differences between wait-list control and intervention groups.

**Table 3:** Effects of the ATTACH^TM^ intervention on parent–child attachment security. [Attachment Improvement scores classified as improved/remained secure (1) or insecure/not improved (0)].

		B	S.E.	Sig.	Exp (B)	95% C.I. for Exp (B)	
						Lower	Upper
Step 1 ^a^	Group 1: Intervention Group	2.293	0.793	**0.004**	0.101	0.021	0.478
	Wave 1 Baseline RCT: PDI-rated RF Overall Score	0.168	0.471	0.721	0.845	0.335	2.129
	Constant	3.894	1.923	0.043	49.087		

^a^ Dependent Variable: Wave 2 Post RCT Pre QES: Attachment Improvement Score. Bold: Statistically significant differences in attachment improvement scores between the wait-list control and intervention groups.

**Table 4:** Effects of the ATTACH^TM^ intervention on parental reflective function.

Effects of the ATTACH^TM^ Intervention on Reflective Function (PDI-Rated RF Self Scores)						
Model		Unstandardized Coefficients		Standardized Coefficients	*t*	Sig.
		B	Std. Error	OR		
1 ^a^	(Constant)	4.086	0.945		4.322	0.000
	Group 1: Intervention Group	1.216	0.398	0.442	3.055	**0.004**
	Wave 1 Baseline RCT: PDI-rated RF Overall Score	0.365	0.238	0.222	1.534	0.134
**Effects of the ATTACH^TM^ intervention on Reflective Function** **(PDI-rated RF Child Scores)**						
Model		Unstandardized Coefficients		Standardized Coefficients	*t*	Sig.
		B	Std. Error	OR		
1 ^b^	(Constant)	3.823	0.817		4.680	0.000
	Group 1: Intervention Group	1.297	0.344	0.504	3.773	**0.001**
	Wave 1 Baseline RCT: PDI-rated RF Overall Score	0.407	0.206	0.264	1.976	0.056
**Effects of the ATTACH^TM^ intervention on Reflective Function** **(PDI-rated RF Overall Scores)**						
Model		Unstandardized Coefficients		Standardized Coefficients	*t*	Sig.
		B	Std. Error	OR		
1 ^c^	(Constant)	3.739	0.829		4.507	0.000
	Group 1: Intervention Group	1.185	0.349	0.465	3.394	**0.002**
	Wave 1 Baseline RCT: PDI-rated RF Overall Score	0.428	0.209	0.281	2.049	0.048

^a^. Dependent Variable: Wave 2 Post RCT Pre QES: PDI-rated RF Self Score. ^b^. Dependent Variable: Wave 2 Post RCT Pre QES: PDI-rated RF Child Score. ^c^. Dependent Variable: Wave 2 Post RCT Pre QES: PDI-rated RF Overall Score. Bold: Statistically significant differences in PDI-rated RF (Self, Child, and Overall scores between the wait-list control and intervention groups.

**Table 5:** Effects of the ATTACH^TM^ intervention on reflective function (PDI-rated RF Self, Child, and Overall Scores) within-person comparisons.

Paired Samples Test								
	Paired Differences					*t*	df	Sig. (2-Tailed)
	Mean	Std. Deviation	Std. Error Mean	95% Confidence Interval of the Difference				
				Lower	Upper			
Baseline PDI-rated RF Self Score Wave 1 RCT Wave 2 QES—Change PDI-rated RF Self Post: Wave 2 RCT Wave 3 QES	0.91	1.30	0.20	1.32	0.49	4.43	39	**0.000**
Baseline PDI-rated RF Child Score Wave 1 RCT Wave 2 QE—Change PDI-rated RF Child Post: Wave 2 RCT Wave 3 QES	1.01	1.07	0.16	1.35	0.66	5.97	39	**0.000**
Baseline PDI-rated RF Overall Score Wave 1 RCT Wave 2 QE—Change PDI-rated RF Overall Post: Wave 2 RCT Wave 3 QES	0.94	1.08	0.17	1.29	0.59	5.48	39	**0.000**

Bold: Statistically significant difference in the PDI-rated RF (Self, Child, and Overall).

## Data Availability

Requests to access the data supporting the findings can be directed to LA, lanis@ucalgary.ca.
